# Light-driven oxygen evolution from water oxidation with immobilised TiO_2_ engineered for high performance

**DOI:** 10.1038/s41598-021-99841-5

**Published:** 2021-10-29

**Authors:** Maria J. Sampaio, Zhipeng Yu, Joana C. Lopes, Pedro B. Tavares, Cláudia G. Silva, Lifeng Liu, Joaquim L. Faria

**Affiliations:** 1 Laboratory of Separation and Reaction Engineering – Laboratory of Catalysis and Materials (LSRE-LCM), Departamento de Engenharia Química, Faculdade de Engenharia, Universidade do Porto, Rua Dr. Roberto Frias s/n, 4200-465 Porto, Portugal; 2 Clean Energy Cluster, International Iberian Nanotechnology Laboratory (INL), Avenida Mestre Jose Veiga, 4715-330 Braga, Portugal; 3 Departamento de Química, Centro de Química-Vila Real, Universidade de Trás-os-Montes e Alto Douro, 5001-911 Vila Real, Portugal

**Keywords:** Engineering, Chemical engineering

## Abstract

Calcination treatments in the range of 500–900 °C of TiO_2_ synthesised by the sol–gel resulted in materials with variable physicochemical (i.e., optical, specific surface area, crystallite size and crystalline phase) and morphological properties. The photocatalytic performance of the prepared materials was evaluated in the oxygen evolution reaction (OER) following UV-LED irradiation of aqueous solutions containing iron ions as sacrificial electron acceptors. The highest activity for water oxidation was obtained with the photocatalyst thermally treated at 700 °C (TiO_2_-700). Photocatalysts with larger anatase to rutile ratio of the crystalline phases and higher surface density of oxygen vacancies (defects) displayed the best performance in OER. The oxygen defects at the photocatalyst surface have proven to be responsible for the enhanced photoactivity, acting as important active adsorption sites for water oxidation. Seeking technological application, water oxidation was accomplished by immobilising the photocatalyst with the highest OER rate measured under the established batch conditions (TiO_2_-700). Experiments operating under continuous mode revealed a remarkable efficiency for oxygen production, exceeding 12% of the apparent quantum efficiency (AQE) at 384 nm (UV-LED system) compared to the batch operation mode.

## Introduction

Oxygen is used in numerous industrial processes, for example, synthesis of chemicals, petroleum processing, glass, ceramic, pulp and paper manufacture. It is mainly used to enhance the reaction rates and to ensure the complete oxidation of possible undesired by-products occurring during some processes^1^. Unfortunately, the transport and delivery of gaseous molecular oxygen pose several treads to its industrial application. Production in situ is undoubtedly an alternative forwarded by many technological strategies.

Therefore, the demand for oxygen production has encouraged the scientific community to expand the concept of artificial photosynthesis that mimics nature to convert solar energy into value-added products. Since the pioneering work of Fujishima and Honda in 1972^2^, reporting the development of a photoelectrochemical cell for the decomposition of water into hydrogen and oxygen under UV light irradiation using the optical semiconductor titanium dioxide (TiO_2_), research in this field became an important topic both for environmental decontamination and energy conversion. A variety of optical semiconductors (e.g., TiO_2_, zinc oxide (ZnO), tungsten trioxide (WO_3_), graphitic carbon nitride (g-C_3_N_4_), bismuth-based materials, black phosphorus, and others) have been employed in several photoactivated applications, including degradation of domestic and industrial sewage, and conversion of natural resources, such as carbon dioxide, nitrogen, and water to value-added chemical and fuels^3–10^.

Concerning water splitting using semiconductor photocatalysis, the process consists of two half-catalytic redox reactions, i.e., the hydrogen evolution reaction (HER) and the oxygen evolution reaction (OER), which are described by the following Eqs. (1) and (2), respectively:

1 $$2{H}^{+}+{2e}^{-}\to {H}_{2}$$

2 $$2{H}_{2}O+4{h}^{+}\to {O}_{2}+4{H}^{+}$$

The OER is viewed as a bottleneck for the energy conversion due to its slow kinetics^11^, which involves four proton-coupled electron transfer steps. These elementary stages (i.e., charge carrier generation, separation and migration) occur during the photocatalytic water oxidation, followed by the surface catalytic reaction. Many efforts have been pursued on developing photocatalysts to accelerate the water oxidation, such as junction construction of metal oxides and by metal deposition, aiming to decrease the energy barrier^10,12^. Despite the significant advances in photocatalysts development neat TiO_2_ continues to receive particular attention due to the nature of the physical interactions between anatase and rutile crystalline phases, which may result in unique trap states and therefore can have a significant impact on the interparticle carrier transport^13^.

Although the catalyst nature is of high importance concerning the water splitting systems, the major limitation is its relatively low efficiency in the presence of pure water, requiring the addition of sacrificial agents. Thus, many examples exist of sacrificial molecules as electron donors to improve hydrogen production in solar-driven water splitting systems^14–16^. However, only a limited variety of electron acceptors have been considered concerning OER. The frequently used electron acceptors are silver cations (Ag^+^), yet some problems arise from their use, i.e., generally, the photocatalytic formation of molecular oxygen is accompanied by the deposition of metallic silver (Ag^0^) nanoparticles, causing irreversible optical changes on the semiconductor properties. Slightly, ferric ions (Fe^3+^) have been employed as sacrificial electron acceptors to produce oxygen^17,18^ owing to its lower redox potential in water (+ 0.77 V; vs. NHE) over Ag^+^ (+ 0.80 V; vs. NHE), and due to the reversible redox couple Fe^3+^/Fe^2+^ in the aqueous phase compared with the Ag^+^ ions. The influence of Fe^3+^ ions as electron acceptors was documented by Matsumura group^17^. In this study, the authors used a TiO_2_ rutile material for water oxidation and found that the reaction is favoured by superior adsorption of Fe^3+^ ions on TiO_2_ over the Fe^2+^ ions.

Therefore, due to the crucial importance of O_2_ in various processes this work explores in situ oxygen production by water oxidation based on semiconductor photocatalysis technology. The influence of oxygen defects, optical and crystalline properties of TiO_2_ samples calcined at different temperatures was investigated for oxygen evolution using a low concentration of Fe^3+^ aqueous solution rather than the typical electron acceptor (Ag^+^). The lower price, high abundance and broad availability of iron compared to silver, the use of Fe^3+^/Fe^2+^ in low concentrations, makes this a cost-effective and sustainable process. In addition, considering the advantages of using continuous mode systems, a selected TiO_2_ sample was deposited in a glass support, and the rate of oxygen production was assessed. To the best of our knowledge, this is the first study on photocatalytic water oxidation using immobilised TiO_2_ towards in situ oxygen production under continuous mode, avoiding the need of recovery the catalyst as an additional step.

## Results and discussion

**Figure 1:**
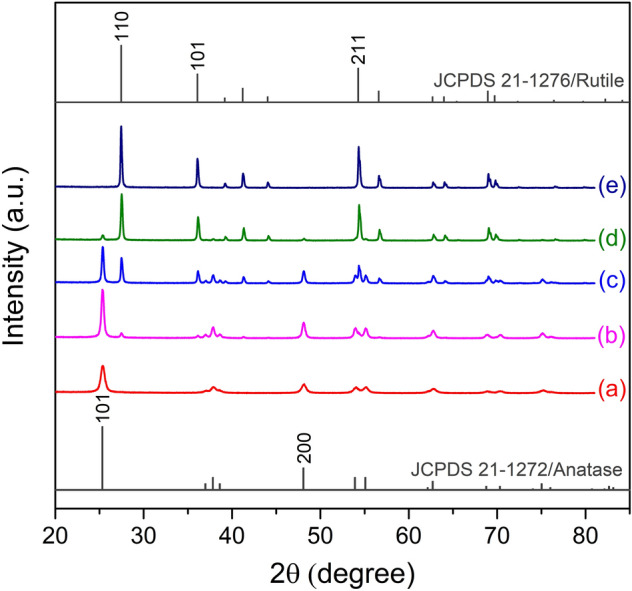
XRD diffractograms of TiO_2_ samples: TiO_2_-500 (**a**), TiO_2_-600 (**b**), TiO_2_-700 (**c**), TiO_2_-800 (**d**) and TiO_2_-900 (**e**).

X-ray diffraction (XRD) analysis was performed to determine the crystalline phase and crystallite size of TiO_2_ samples (Fig. 1, Table 1). The peaks appearing at 25° and 48°, correspond to the lattice planes of 101 and 200 (JCPDS 21-1272) of TiO_2_ anatase phase. The peaks at 27°, 36° and 54° with lattice planes of 110,101 and 211 (JCPDS 21-1276), respectively reveal the presence of rutile phase. As displayed in Fig. 1 the diffraction peaks of both anatase and rutile phases of TiO_2_ samples become intense as the calcination temperature increases, suggesting that TiO_2_ samples are composed of irregular polycrystalline materials, and the crystallinity for both phases is improved by rising the temperatures.

**Table 1:** Crystallisation parameters, specific surface area (S_BET_), bandgap and oxygen defects of TiO_2_ samples.

Sample	Anatase (A)/rutile (R) ratio (%)	Crystallite size (nm) (A)	Crystallite size (nm) (R)	S_BET_ (m^2^ g^−1^)	Bandgap (eV)	Oxygen defects (%)
TiO_2_-500	–	19.4	–	36.8	3.28	16.8
TiO_2_-600	57.8	17.4	30.1	24.6	3.27	24.5
TiO_2_-700	88.8	43.7	49.2	17.1	3.13	31.7
TiO_2_-800	73.1	25.3	34.6	8.3	3.08	30.7
TiO_2_-900	–	–	43.7	6.2	3.05	26.1

**Figure 2:**
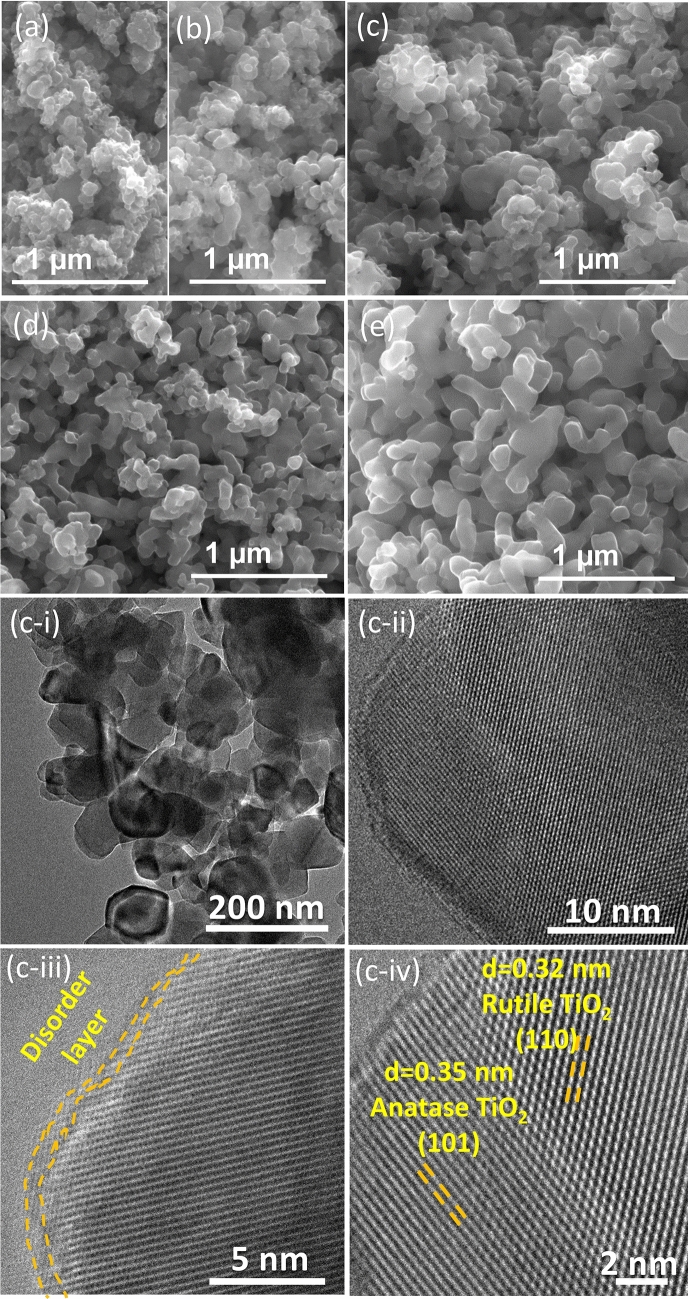
SEM images of TiO_2_ samples: TiO_2_-500 (**a**), TiO_2_-600 (**b**), TiO_2_-700 (**c**), TiO_2_-800 (**d**) and TiO_2_-900 (**e**). Representative TEM and HRTEM images (**c-i,ii,iii,iv**) of TiO_2_-700 taken at different magnifications.

Representative SEM images were obtained to visualise the morphology of the different TiO_2_ samples (Fig. 2). Results reveal an irregular shape of TiO_2_ particles with the increase of the calcination temperature. For TiO_2_-500 and TiO_2_-600 (Fig. 2a,b), spherical-like TiO_2_ particles are observed with distinct sizes aggregate into clusters. At temperatures over 700 °C, it seems that small particles coalesce to form larger particles (Fig. 2c), due to the transformation of anatase into rutile phase (Fig. 1). At 900 °C the transformation of anatase–rutile is completed and agglomerations of more a uniform size of TiO_2_ particles are noticed (Fig. 1e).

The TEM and high-resolution transmission electron microscopy (HRTEM) were used to further investigate the microstructure and composition of representative TiO_2_ sample (TiO_2_-700, Fig. 2c). In this case, the particles show a typical size of ca. 100 nm, for its crystallized phase (Fig. 1c-i,c-ii, respectively). In Fig. 2c-iii, the disordered layers on the surface of the TiO_2_-700 sample are visible and marked with orange dashed lines, suggesting the presence of defects that can provide higher carrier concentration and more active sites^19^.

**Figure 3:**
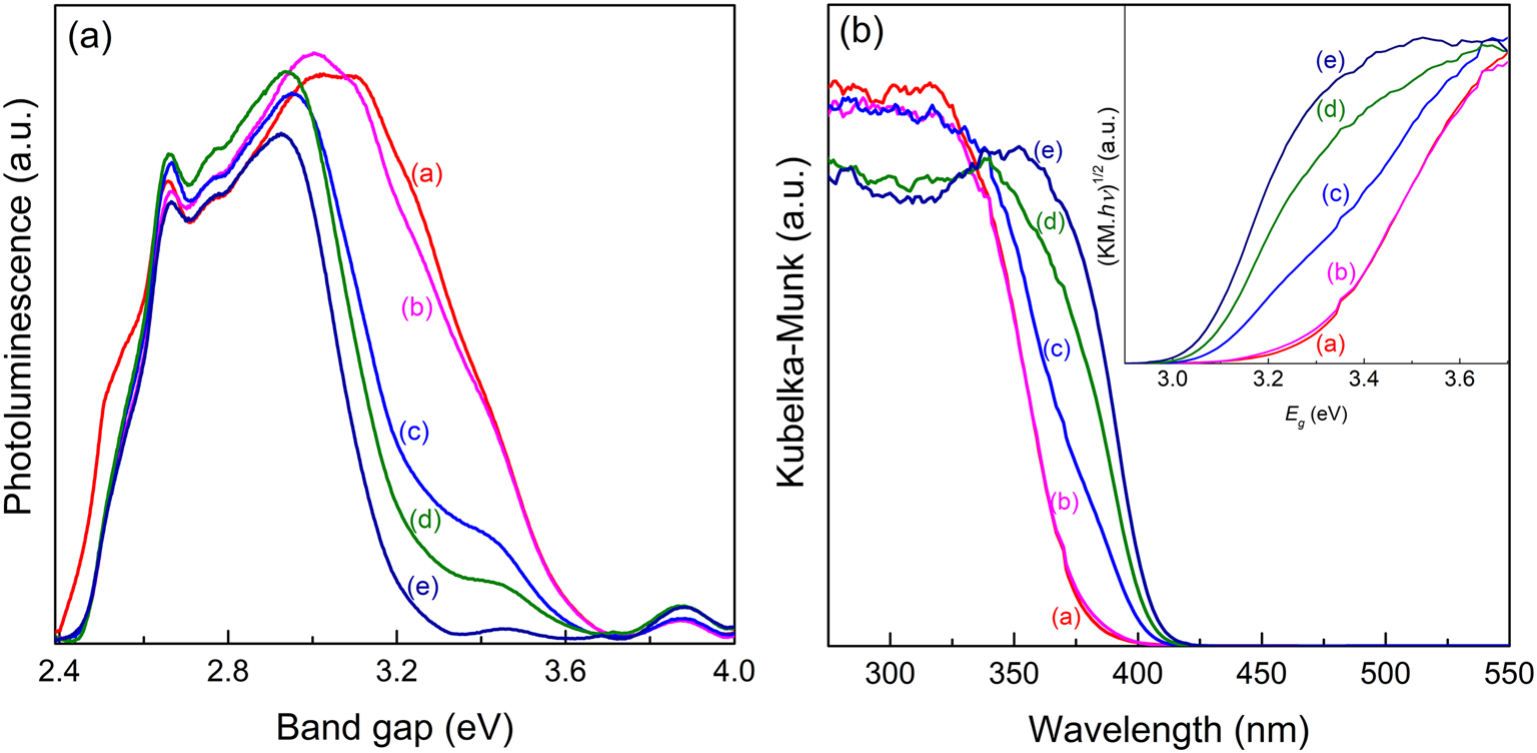
Photoluminescence (PL) spectra of TiO_2_ samples (**a**), and DRUV-Vis and Tauc Plot (inset) for indirect bandgap (**b**): TiO_2_-500 (a), TiO_2_-600 (b), TiO_2_-700 (c), TiO_2_-800 (d) and TiO_2_-900 (e).

HRTEM image revealed well-resolved crystal lattices in a representative TiO_2_-700 (Fig. 3c-iv), and the inter-planar spacing values of 0.32, 0.35 nm correspond to the (110) and (101) crystal planes of rutile and anatase TiO_2_, respectively, verifying the co-existence of rutile and anatase in a single nanoparticle, consistent with the XRD results.

Specific surface areas of TiO_2_ samples were determined by nitrogen adsorption at 77 K (Table 1). As expected, the results show a decrease of S_BET_ with the enhancement of the calcination temperature, agreeing with the SEM observations.

The TiO_2_ samples were examined by PL spectroscopy at room temperature under an excitation energy of 4.43 eV (280 nm) to investigate the incidence of charge separation upon light excitation (Fig. 3). The intense PL emission in the UV and visible regions is commonly attributed to excitons recombination and the presence of defect sites, respectively^20^. The samples TiO_2_-500 (Fig. 3a) and TiO_2_-600 (Fig. 3b) show a broad peak at 3.0 eV ascribed to the crystallinity of TiO_2_. By increasing the annealing temperature (samples c, d and e), a noticeable red shift is observed for 2.93 eV, suggesting that the TiO_2_-700, TiO_2_-800 and TiO_2_-900 samples exhibited higher crystallinity, as confirmed by HRTEM observations (Fig. 2). Additionally, these strong peaks (2.93 and 3.00 eV) have been ascribed to the self-trapped excitons in anatase and free excitons in rutile, respectively^20^. The peaks at 2.63–2.82 eV arise from the excitation electrons/holes recombination via oxygen vacancies and defects in both anatase and rutile phases of TiO_2_.

The diffuse reflectance spectra of the TiO_2_ samples were performed (Fig. 3b) and the respective bandgap values were obtained by indirect Tauc plot analysis (Table 1). As displayed, increasing the calcination temperature the bandgap of TiO_2_ samples slightly diminish, which may indicate faster electron/hole recombination rates^21^.

**Figure 4:**
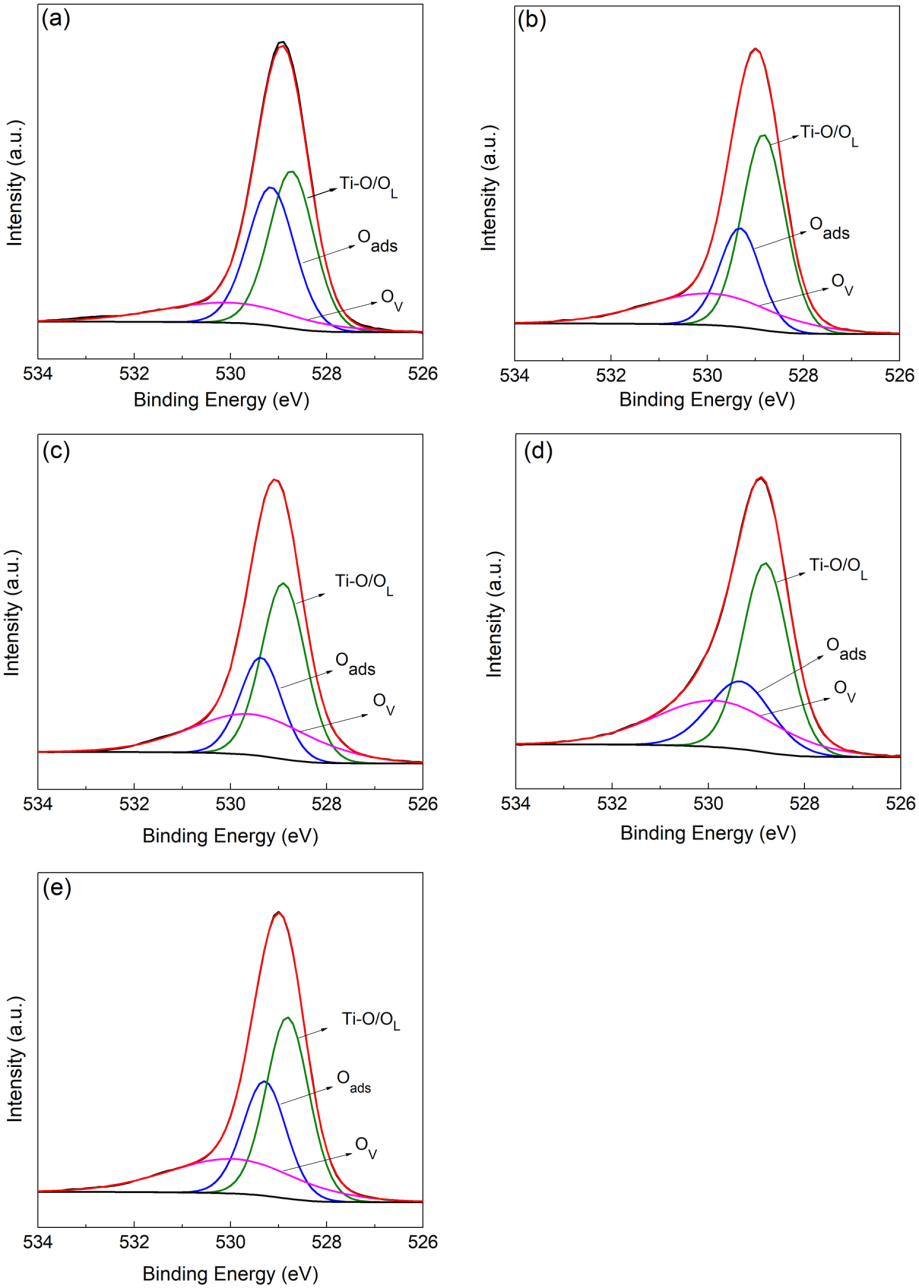
XPS spectra of the TiO_2_ samples: TiO_2_-500 (**a**), TiO_2_-600 (**b**), TiO_2_-700 (**c**), TiO_2_-800 (**d**) and TiO_2_-900 (**e**). O_v_, O_L_ and O_ads_ correspond to oxygen vacancies, lattice oxygen and surface chemisorbed hydroxyl groups, respectively.

We further have examined the surface chemical states of TiO_2_ using XPS spectroscopy. Given that XPS allows investigating the surface atomic constituents, it has been used to characterise the degree of oxygen defect according to the relative element contents, and the intensities and positions of peaks^22,23^. Among the defects identified in TiO_2_, the presence of oxygen vacancies (O_v_) can act as active adsorption sites, and strongly influence the reactivity of the photocatalysts^22^. Additionally, the formation of O_v_ commonly leads to the creation of unpaired electrons that can generate donor levels in the electronic structure of the TiO_2_^24,25^. As observed in Fig. 4 the O 1s XPS spectra of the TiO_2_ samples were deconvoluted into three main peaks at 528.8 eV, 529.3 eV and 529.9 eV energies. The peak located at 528.8 eV is assigned to Ti–O–Ti bonding and lattice oxygen (O_L_) within the TiO_2_ structure^24^. In contrast, the peaks at 529.3 eV and 529.9 eV energies are associated to surface chemisorbed hydroxyl groups (O_ads_) and O_v_, respectively. As displayed in Table 1, the area proportional to oxygen defects increase with the calcination temperature of TiO_2_ samples until 700 °C (TiO_2_-500, TiO_2_-600, and TiO_2_-700; 16.8%, 24.5%, and 31.7% respectively). For highest calcination temperatures (TiO_2_-800, TiO_2_-900; 30.7% and 26.1%, respectively) the oxygen defects start to decrease, suggesting that surface O_v_ concentration gradually diminishes with temperatures above 700 °C, being consistent with the literature^24,26^.

### Oxygen evolution reactions

Photocatalytic oxygen evolution was evaluated by TiO_2_ samples calcined at various temperatures under UV-LED radiation using an aqueous Fe(NO_3_)_3_·9H_2_O solution acting as sacrificial electron acceptor. Although the mechanism of O_2_ evolution using silver cations (Ag^+^) as electron acceptors is well known in the literature^15,27^ the photocatalytic generation of molecular oxygen is followed by the deposition of metallic silver nanoparticles on the surface of the photocatalyst. Irreversible reactions accompany this phenomenon due to the plasmonic adsorption band of the silver particles^15^. Due to this issue and the high documented studies using Ag^+^ ions as sacrificial agent in photocatalytic water oxidation preliminary experiments were performed under the same operational conditions using this electron acceptor for comparison purposes (Supplementary Fig. S1). The results revealed a slight increasing efficiency for O_2_ production in the first 30 min using Ag^+^ ions solution compared with the Fe^3+^. However, with the reaction time a progressive reduction of Ag^+^ into Ag^0^ and the oxygen evolution rates decreases.

**Figure 5:**
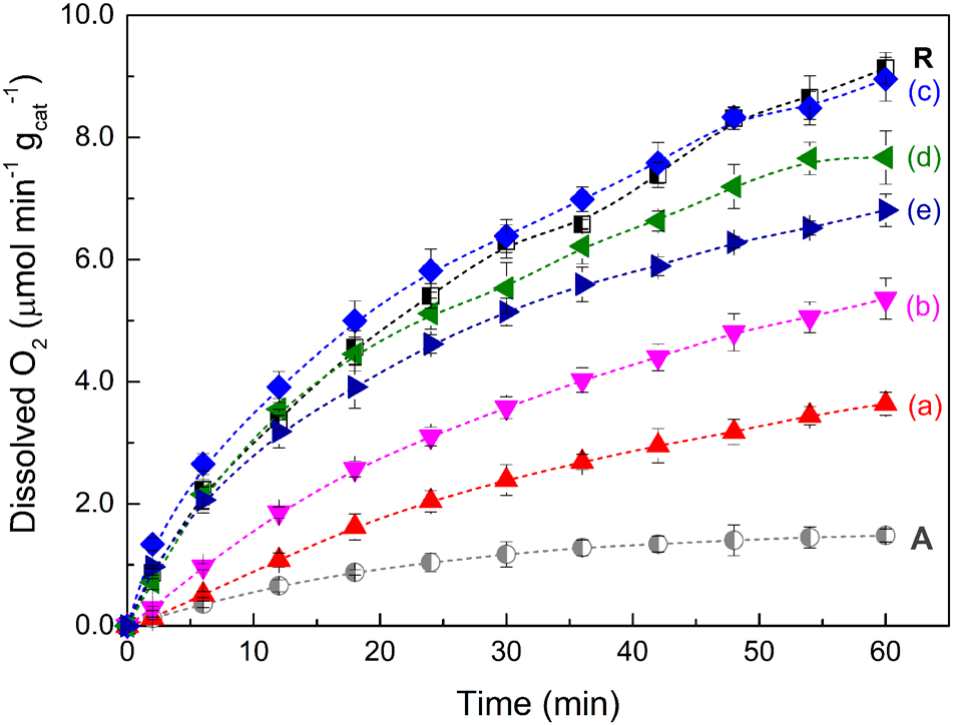
Photocatalytic dissolved oxygen evolution using TiO_2_-500 (**a**), TiO_2_-600 (**b**), TiO_2_-700 (**c**), TiO_2_-800 (**d**) and TiO_2_-900 (**e**); n = 3 (standard deviation < 5%). R and A correspond to commercial TiO_2_ 100% rutile and 100% anatase, respectively.

Concerning the blank experiments (i.e., dark conditions, absence of catalyst and sacrificial agent), using a Fe^3+^ aqueous solution no formation of O_2_ was noticed. Contrary, photocatalytic water oxidation reactions revealed that all the tested samples were found to generate O_2_ upon UV-LED irradiation, although the efficiencies were dependent on the calcination temperature of TiO_2_ samples (Fig. 5). In general, the results show that when the calcination temperature is raised, the efficiency of the photocatalytic process is enhanced. Nevertheless, after a specific temperature (800 °C), a decrease in O_2_ evolution was observed. Among the photocatalysts, the TiO_2_-700 sample showed the best performance for water oxidation with 8.95 µmol min^−1^ g_cat_^−1^ of dissolved oxygen being detected.

The calcination treatment commonly affects the physicochemical properties of optical semiconductors, such as crystalline phase and crystallite size, specific surface area, and oxygen surface defects. Therefore, it is commonly accepted that a higher specific surface area (S_BET_) promotes a significant number of active adsorption sites and improves reactivity. Yet, from the results it is unlikely that the efficiency of the TiO_2_ samples is related to their specific surface area once was found a decrease in the S_BET_ with the rise of the calcination temperature. This diminution on the S_BET_ was more noticeable for the samples calcined at 800 and 900 °C due to the enhancement of the TiO_2_ particles size as observed by SEM micrographs (Fig. 2). Additionally, the presence of higher percentage of rutile phase in these samples may indicate faster electron/hole recombination, which may explain the decrease in the efficiency of the water oxidation process^24^.

**Figure 6:**
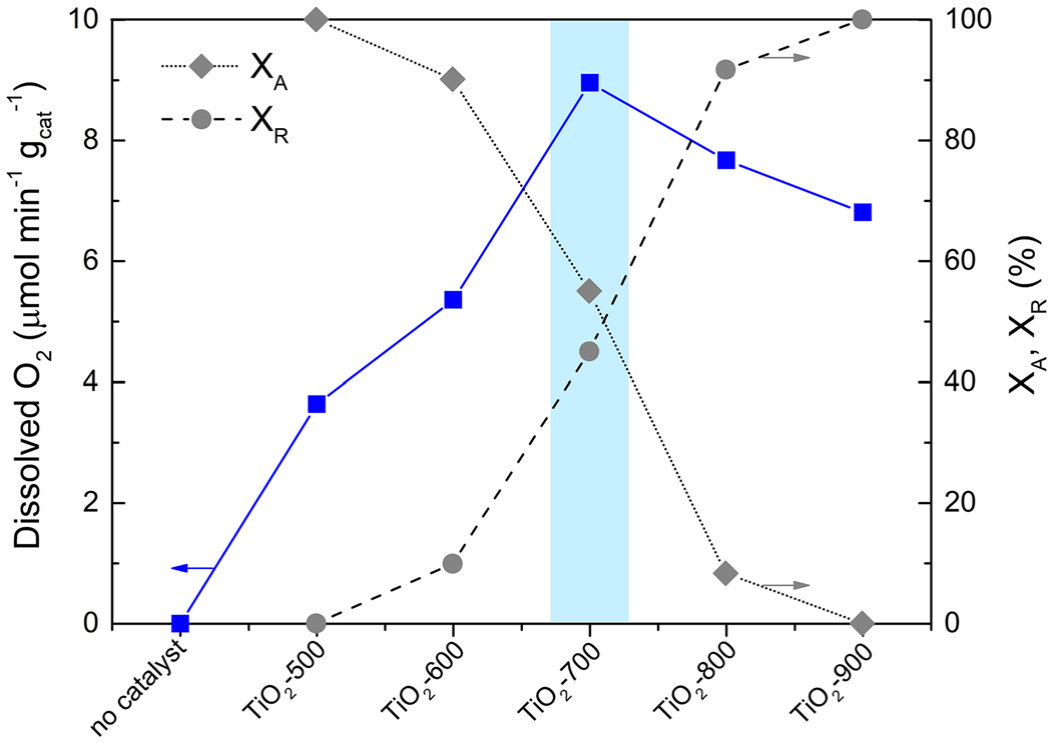
Photocatalytic dissolved oxygen evolution using TiO_2_ samples calcined at different temperatures (left side), and percentage of anatase and rutile phases (right side).

Although in terms of oxygen evolution, effect of the particle size and the crystallite phases of the photocatalysts is not clearly understood, some reports suggest that these factors may contribute to the efficiency of photocatalytic water oxidation. Hiroshi et al.^28^, ascribed the performance of the water oxidation photocatalytic process to the crystallization from anatase to rutile phase. More recently, Maeda et al.^29^ have attributed the high efficiency of TiO_2_ catalysts for oxygen evolution to the enhancement of the particle size which seems to hinder the electron/hole recombination. Higher efficiencies for O_2_ production have been assigned to the TiO_2_ rutile phase unlike what happens in the degradation of organic compounds using TiO_2_ anatase^30,31^. To understand the efficiency of the developed TiO_2_ samples in terms of crystallization, experiments using commercial 100% TiO_2_ anatase and 100% rutile were tested under the same experimental conditions (Fig. 5; A and R, respectively). As shown, high efficiency was found with the commercial TiO_2_ rutile. Nevertheless, for the synthesised TiO_2_ calcined at 900 °C 100% rutile (Table 1) the performance for O_2_ production was lower compared with the TiO_2_-700 and also with TiO_2_-800 samples, suggesting that the other factors account to the efficiency of the process using the TiO_2_ samples prepared by sol–gel method. As documented, the ratio between surface anatase and rutile particles increases the photocatalytic activity in water splitting reactions^32,33^. As displayed in Fig. 6, the performance of the photocatalytic water oxidation for O_2_ evolution was achieved when the ratio between anatase and rutile crystalline phases are superior (88%, Table 1), corroborating with the reported investigations^33,34^.

It is recognised that lattice defects such as oxygen vacancies (O_v_) can provide intermediate band levels or additional states into the forbidden band of the semiconductor that may trap electrons, which can increase the light absorption capacity and charge separation^29,35^. Moreover, the high density of O_v_ (electron donors) is thought to shift the Fermi level of photocatalysts such as TiO_2_ near the conduction band, enabling the charge separation at the TiO_2_ particle interface, thus enhancing the degree of band bending at the semiconductor surface. Although the thermal treatments are known to increase the density of O_v_, above a certain temperature, the number of O_v_ is reduced^19,24,29^. Amano et al.^33^ investigated the photocatalytic activity of a TiO_2_ commercial sample treated at 700 °C under H_2_ flow. The authors ascribed the photocatalytic performance to an increase in the density of the oxygen vacancies at the photocatalyst surface, which enhanced the electrons availability in the conduction band.

Regarding the characterisation results, it can be inferred that the high reactivity of the TiO_2_-700 sample for water oxidation is due to the higher percentage of oxygen vacancies at the catalyst surface as was found by XPS analysis. This higher density of oxygen vacancies may facilitate the charge carrier diffusion in TiO_2_, improving the photocatalytic activity for O_2_ production. Although, the percentage of oxygen vacancies in the TiO_2_-800 sample (30.7%) is negligible compared with the TiO_2_-700 sample (31.7%), a decrease in the rutile particle size was observed (Table 1). The same behaviour was detected for TiO_2_-900, i.e., smaller rutile particle size than the TiO_2_-700, suggesting that excess thermal treatment is being used to decrease the oxygen vacancies, leading to the photocatalytic decreasing performance, being reliable with literature^26^. Additionally, tries to obtain electrochemical evidence of the oxidation potential of the photocatalysts were performed by supporting the TiO_2_ samples on conductive ITO electrode. The TiO_2_-700 and TiO_2_-800 samples (Supplementary Fig. S2) revealed an oxidation peak between 0.5 and 0.7 V vs SCE, suggesting that positive holes can be generated in the photocatalysis process, compatible with the observed results for photocatalytic oxygen production^36^.

**Scheme 1:**
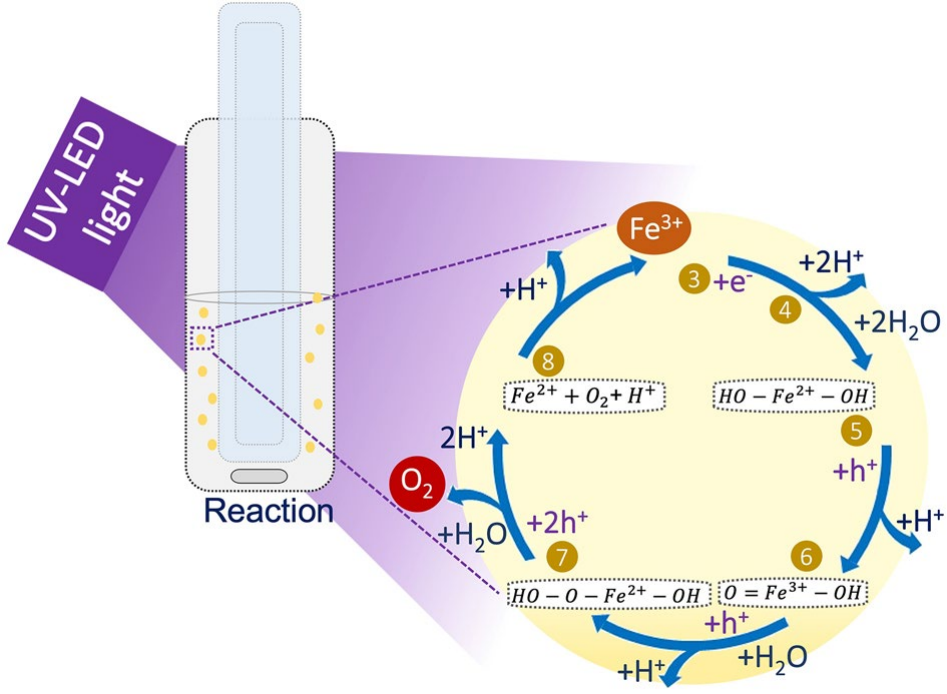
Schematic representation of a proposed mechanism for O_2_ evolution from Fe(NO_3_)_3_·9H_2_O water solution under UV-LED light irradiation using TiO_2_ photocatalysts. The numbers from 3 to 8 pair with Eqs. (3)–(8).

As described above, a significant number of investigations have been devoted to understanding the photocatalytic water oxidation using Ag^+^ cations as sacrificial electron acceptors, yet, using Fe^3+^ aqueous solutions few reports have been documented. Concerning the results obtained with the TiO_2_ samples using the Fe^3+^ ion solutions it seems reasonable to propose that the overall process is governed by the mechanism illustrated in Scheme 1, and by the following equations:

3 $${Fe}^{3+}+{e}^{- }\to {Fe}^{2+}$$

4 $${Fe}^{2+}+2{H}_{2}O\to {(HO-Fe}(II)-OH)+2{H}^{+}$$

5 $${(HO-Fe}(II)-OH)+{h}^{+}\to (O={Fe}(III)-OH)+{H}^{+}$$

6 $$(O={Fe}(III)-OH)+{H}_{2}O+{h}^{+}\to {(HO-O-Fe}(II)-OH)+{H}^{+}$$

7 $$(HO-{O-Fe}(II)-OH)+{H}_{2}O+{2h}^{+}\to {(HO-Fe}(II)-OH)+{2H}^{+}+{O}_{2}$$

Additionally, as the O_2_ is being accumulated in the reaction system the oxidation of Fe^2+^ into Fe^3+^ may also occur (Eq. (8)). Although in practice the oxidation in acid media is hard, at a moderate pH the iron(III) oxide hydrate (Fe_2_O_3_) can precipitate, and a reduction reaction of Fe^3+^ to Fe^2+^ can take place quickly (Eq. (9)). As a result, hydrogen peroxide (HOOH) could be formed, occurring again the oxidation of Fe^2+^ into Fe^3+^ (Eq. (10)). Nevertheless, this last hypothesis seems to be challenging to occurs, as the maximum pH measured during the reactions was ca. 2.28.

8 $${Fe}^{2+}+{O}_{2}+{H}^{+}\to {Fe}^{3+}+\left(HO-{O}^{\bullet }\right)$$

9 $$(HO-{O}^{\bullet })+{Fe}^{3+}+{H}^{+}\to HOOH+{Fe}^{2+}$$

10 $$HOOH+{Fe}^{2+}+{H}^{+}\to {HO}^{\bullet }+{H}_{2}O+{Fe}^{3+}$$

Although the set of Eqs. (3)–(7) seems to describe the results obtained with the TiO_2_ samples, a maximum amount of oxygen was evaluated after 60 min in the reaction conditions; after that, the reaction system achieved the steady state (Supplementary Fig. S3). This can be ascribed to the reaction system configuration used, i.e., as the process performance was evaluated in terms of in situ O_2_ generation with the product remaining in reaction, which may indicate the O_2_ solubility in the aqueous media was reached.

Therefore, considering the above results, a potential technological application for oxygen production was assessed using the TiO_2_-700 immobilised in a glass slide and tested under continuous mode (Supplementary Scheme S1b). Using this reactor configuration we can overcome the practical problems that arise from using a catalyst in powder form and ensure continuous feed of sacrificial electron acceptors. The apparent quantum efficiency (AQE) was examined for both reaction systems by the following equation:

$$AQE=\frac{4\times n\times {N}_{A}\times h\times c}{A\times I\times t\times \lambda }$$

where *n* is the amount of O_2_ molecules, *N*_*A*_ the Avogadro constant, *h* the Plank constant, *c* the speed of light in vacuum (m/s), *A* the irradiated area (78.5 and 19.8 cm^2^, batch, and continuous systems, respectively), *I* the exposed irradiation intensity in each system, *t* the time of the reactions (s), and *λ* the wavelength of the monochromatic LED (384 nm).

**Figure 7:**
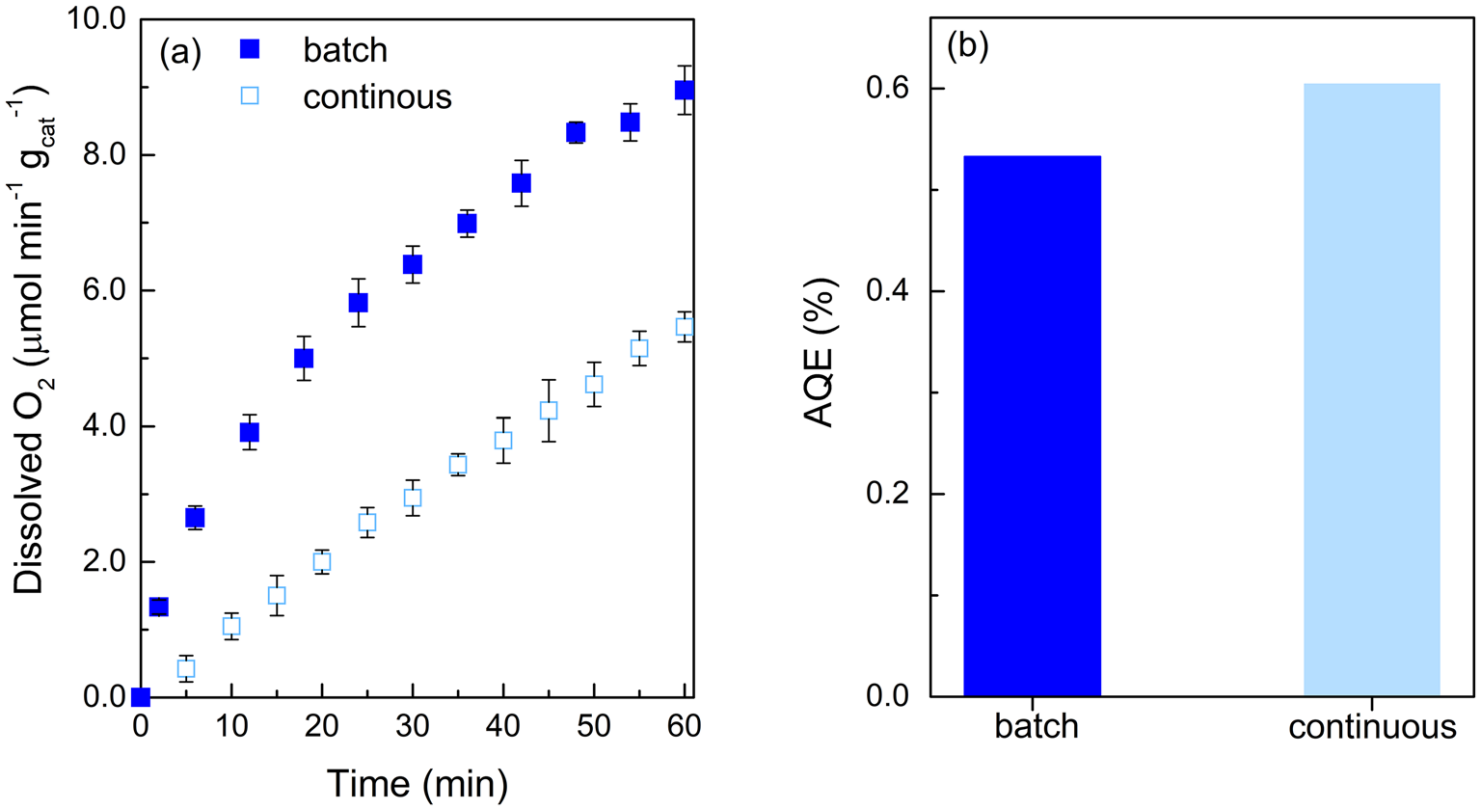
Comparison of oxygen evolution using batch and continuous mode reaction systems (**a**); n = 3 (standard deviation < 5%), and Apparent Quantum Efficiency (AQE) of the both reaction processes (**b**).

As displayed in Fig. 7 a, after 60 min of reaction the concentration of O_2_ is lower operating in continuous mode (5.46 µmol min^−1^ g_cat_^−1^) comparing with slurry mode (8.95 µmol min^−1^ g_cat_^−1^). Nevertheless, it is important to refer that a larger amount of catalyst per volume is used in the slurry system (0.120 g) in contrast with the continuous mode (0.013 g). Nevertheless, the results reveal that the immobilisation of TiO_2_-700 as film enables a remarkable efficiency for O_2_ production, exhibiting AQE exceeding 12% (Fig. 7b) at 384 nm compared with the slurry mode. This configuration system may constitute a step forward for developing efficient and viable systems capable of producing O_2_ in continuous-flow reactors, reducing the operating costs and allowing large-scale production.

## Conclusions

TiO_2_ photocatalysts were synthesised by the sol–gel method and successfully used for water oxidation. High photocatalytic performance for oxygen evolution was achieved using iron nitride aqueous solution as sacrificial electron acceptors. Among the photocatalyst studied, the TiO_2_-700 material revealed the highest efficiency with an in situ oxygen production rate of 8.95 µmol min^−1^ g_cat_^−1^ being determined. The results were correlated with the physicochemical and morphological properties of the developed TiO_2_ photocatalysts. The specific surface area of the resulting photocatalysts was found negligible for O_2_ production, as well as the bandgap of the materials. The remarkable efficiency of the TiO_2_-700 for oxygen evolution was correlated with the highest ratio between anatase and rutile phases (88%), which seems to indicate an efficient separation of the photogenerated electron/hole carriers. In addition, the superior reactivity of the TiO_2_-700 sample was ascribed to the enhancing of the oxygen vacancies at the photocatalyst surface, which act as active sites for water oxidation.

Finally, the TiO_2_-700 was successfully immobilised over a glass support for a continuous production of oxygen. The continuous-flow reaction system demonstrated promising results for water oxidation revealing a superior AQE at 384 nm compared with the results obtained in the batch system, 0.60 and 0.53, respectively.

## Materials and methods

TiO_2_ rutile (99%) was purchased from Iolitec. Iron(III) nitrate nonahydrate (≥ 98%; Fe(NO_3_)_3_·9H_2_O), titanium butoxide (97%; Ti(OCH_2_CH_2_CH_2_CH_3_)_4_), TiO_2_ anatase (> 99%) and citric acid (≥ 99.5%; HOC(COOH)(CH_2_COOH)_2_) were supplied from Sigma-Aldrich. Absolute Ethanol (CH_3_CH_2_OH) was purchase from VWR. Ultrapure water was obtained from a Milli-Q water system (resistivity of 18.2 MΩ cm, at 25 °C).

### Catalyst preparation and characterisation

Titanium dioxide samples were prepared from the sol–gel method: a solution with ethanol:titanium butoxide (50:3.5; molar ratio) was added drop-by-drop to a solution containing water:ethanol:citric acid (50:60:0.4; molar ratio) under continuous stirring. The final solution was kept mixed for 30 min followed by ageing in the air for several days, then the resulting xerogel was ground and dried at room temperature. The powders were calcined under the air atmosphere. Briefly, 1 g of powder was placed in a quartz crucible in a Microwave Phoenix apparatus (CEM Corporation) under static air conditions for 90 min at a given temperature. The final catalysts were designed as TiO_2_-T, where T corresponds to the calcination temperature (between 500 and 900 °C).

Uncoated glass supports (7.6 × 2.6 cm) were extensively cleaned under sonication with an anionic detergent in water, followed by the immersion in 2-propanol for 15 min under sonication, and finally dried. A paste of a selected TiO_2_-T sample was prepared as described in our previous work^37^. The resulting photocatalyst paste was spread on the washed glass slides using the casting knife applicator (Elcometer 3580, Warren, MI). Then, the TiO_2_-coated slides were calcined at 500 °C in static air for 3 h. The amount of catalyst immobilised was found from the difference between uncoated and coated slides after the calcination treatment.

X-ray diffraction (XRD) measurements were conducted in a PANalytical X’Pert MPD equipped with an X’Celerator detector and secondary monochromator (Cu Kα *λ* = 0.154 nm, 40 kV,30 mA).

X-ray photoelectron spectroscopy (XPS) characterisation was carried out on an ESCALAB 250 instrument with Al Kα X-rays (1486.6 eV).

The morphology of the photocatalysts was examined by scanning electron microscopy (SEM) using a FEI Quanta 400 FEG ESEM/EDAX Genesis X4M (15 keV) microscope. High-resolution transmission electron microscopy (HRTEM) was conducted on a probe-corrected transmission electron microscope operating at 200 kV (FEI Themis 60–300).

The N_2_ adsorption–desorption isotherms at −196 °C were acquired in a Quantachrome NOVA 4200e device. The Brunauer–Emmett–Teller equation was used to obtain the specific surface area (S_BET_) from the N_2_ adsorption data in the relative pressure range 0.05–0.20 (Supplementary Fig. S4).

Diffuse Reflectance UV–Visible (DRUV-vis) spectra were obtained within the 220–800 nm wavelength range using a JASCO V-560 spectrophotometer equipped with an integrating sphere at room temperature. Photoluminescence (PL) analyses of the powder photocatalysts were attained at room temperature by a JASCO (FP 82000) fluorescence spectrometer with a 150 W Xenon lamp as a light source, using bandwidths of 10 nm for emission and excitation.

### Photocatalytic water oxidation

Water oxidation experiments were conducted in a slurry glass-immersion reactor filled with 120 mL of 5 mM Fe(NO_3_)_3_·9H_2_O solution acting as a sacrificial agent. Preliminary experiments were performed varying the initial concentration of the Fe(NO_3_)_3_·9H_2_O solution between 1.0 and 50.0 mM (Supplementary Fig. S5).The photocatalytic system was maintained at ca. 20 ℃ using a cooling water system. In a typical experiment, TiO_2_ powders were dispersed in the Fe(NO_3_)_3_·9H_2_O solution and the resulting suspension (1 g L^−1^) was completely deaerated by saturation with argon (50 mL min^−1^) for 30 min. Then, the reactor headspace was maintained under the argon atmosphere (10 mL min^−1^) to hinder oxygen gas–liquid transfer through the interface. The oxygen concentration was measured by an O_2_-sensor FOXY probe (Ocean Optics NeoFOX) dipped in the suspensions. The photocatalytic reactor was exposed to a four LEDs system with a maximum wavelength of 384 nm (Supplementary Scheme S1a). The intensity of each LED varied between 120 and 127 W m^−2^, which was measured at 3 cm from the reactor wall by a UV–Vis spectroradiometer apparatus (USB2000+, OceanOptics, USA).

Additional tests under continuous mode were performed using an acrylic reactor system with a maximum volume of 40 mL constituted by a chamber containing the immobilised TiO_2_-700 film (0.013 g). The reactor was sealed and purged with an argon flow (10 mL min^−1^) for ca. 2 h. The irradiation device, constituted by one LED source (λ_max_ = 384 nm; 127 W m^−2^), was used at 3 cm from the immobilised catalyst (Supplementary Scheme S1b). The reactor system was continuously fed with 5 mM of Fe(NO_3_)_3_·9H_2_O solution previously saturated with argon. The residence time was kept at 20 min, taking into account the results obtained in batch mode. The accumulated dissolved oxygen was measured using an O_2_-sensor FOXY probe.

Blank experiments were performed in all experiments, namely in the absence of catalysts, without adding sacrificial agents under illumination and dark conditions. All the reactions were carried out in triplicate.

## Supplementary Information

Supplementary Information.
