# Supplementary material for: Light-driven oxygen evolution from water oxidation with immobilised TiO2 engineered for high performance
Source: Sci Rep. 2021 Oct 29;11:21306. doi: 10.1038/s41598-021-99841-5 (PMC8556285; doi:10.1038/s41598-021-99841-5)
Supplement: Supplementary file 1 — Supplementary Information. [file 41598_2021_99841_MOESM1_ESM.pdf]

Light-driven oxygen evolution from water oxidation with immobilised TiO_2 engineered for high performance

Maria J. Sampaio[1,*], Zhipeng Yu[1,2], Joana C. Lopes[1], Pedro B. Tavares[3], Cláudia G. Silva[1], Lifeng Liu[2], Joaquim L. Faria[1]

[1]*Laboratory of Separation and Reaction Engineering – Laboratory of Catalysis and Materials (LSRE-LCM), Departamento de Engenharia Química, Faculdade de Engenharia, Universidade do Porto, Rua Dr. Roberto Frias s/n, 4200-465 Porto, Portugal*

[2]*Clean Energy Cluster, International Iberian Nanotechnology Laboratory (INL), Avenida Mestre Jose Veiga, 4715-330 Braga, Portugal*

[3]*Centro de Química-Vila Real, Departamento de Química, Universidade de Trás-os-Montes e Alto Douro, 5001-911 Vila Real, Portugal*

[*]*Corresponding author: mjsampaio@fe.up.pt*

Supplementary data

Table of Contents

Figure S1. Photocatalytic dissolved oxygen evolution by TiO_2-700 sample using $AgNO_3$ and $Fe(NO_3)_3.9H_2O$ as electron acceptors.

Figure S2. Cyclic voltammetry of the TiO_2 samples: TiO_2-500 (a), TiO_2-600 (b), TiO_2-700 (c), TiO_2-800 (d) and TiO_2-900 (e) films supported on a FTO electrode immersed in 0.5 M Na_2SO_4 electrolyte recorded under identical conditions.

Figure S3. Comparison of oxygen evolution using batch and continuous mode reaction systems using the TiO_2-700 sample.

Figure S4. N_2 adsorption-desorption isotherms at −196 °C for TiO2 materials.

Figure S5. Concentration profiles of sacrificial electron acceptor ($[Fe(NO_3)_3{\cdot}9H_2O]_0$) (a), and concentration of dissolved oxygen after 30 min reaction using TiO_2-700 (b).

Scheme S1. Photocatalytic experimental set-up under batch reaction mode (a) and a fixed bed reactor operating under continuous mode (b).

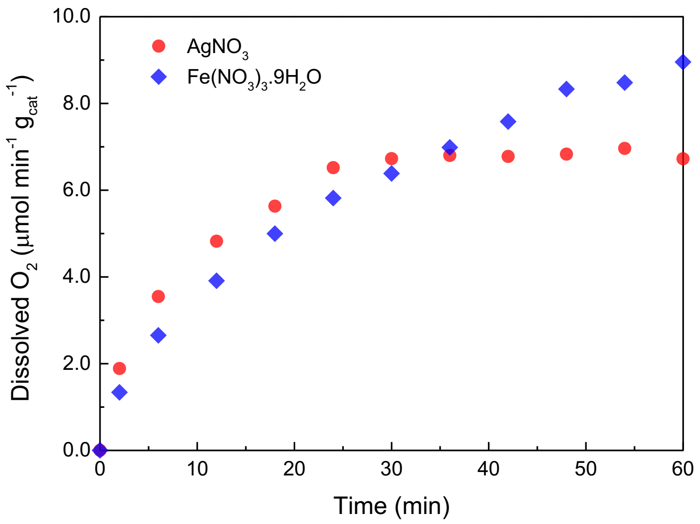

Figure S1. Photocatalytic dissolved oxygen evolution by TiO_2-700 sample using $AgNO_3$ and $Fe(NO_3)_3.9H_2O$ as electron acceptors.

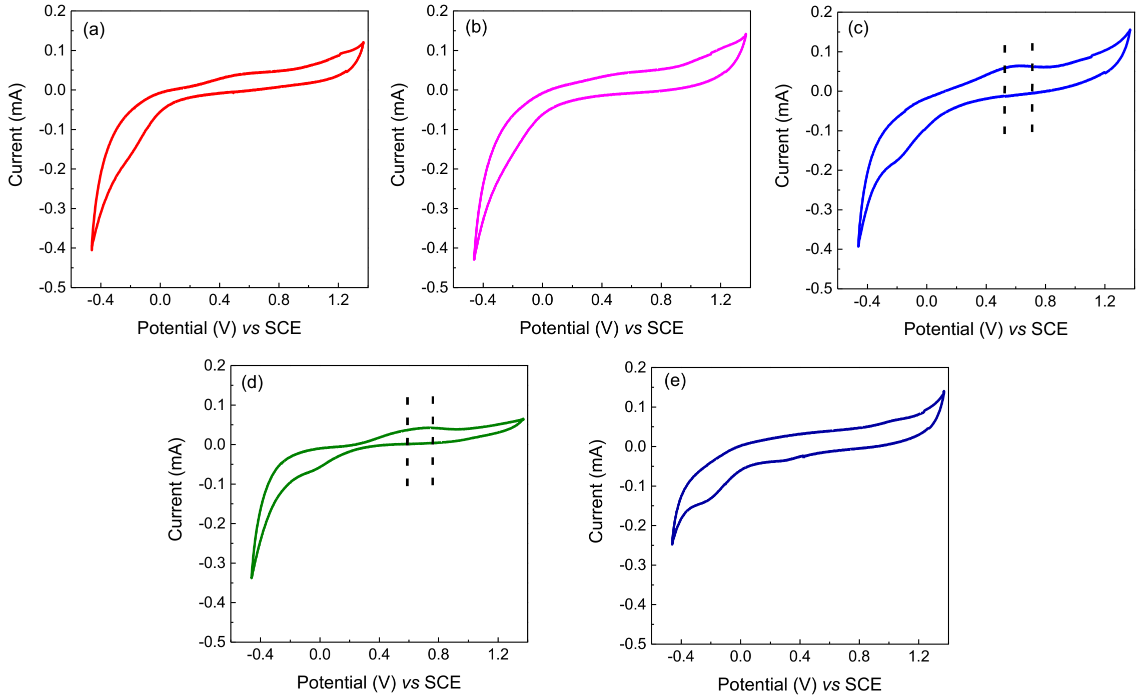

Figure S2. Cyclic voltammetry of the TiO_2 samples: TiO_2-500 (a), TiO_2-600 (b), TiO_2-700 (c), TiO_2-800 (d) and TiO_2-900 (e) films supported on a FTO electrode immersed in 0.5 M Na_2SO_4 electrolyte recorded under identical conditions.

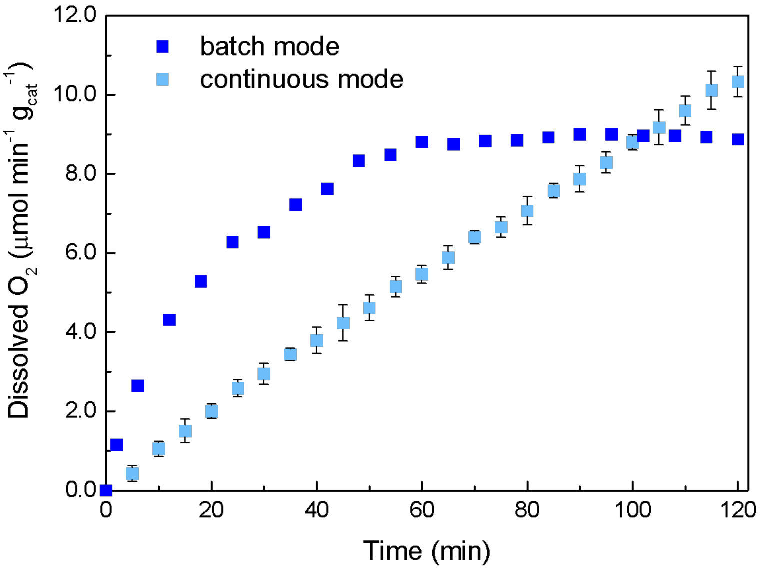

Figure S3. Comparison of oxygen evolution using batch and continuous mode reaction systems using the TiO_2-700 sample.

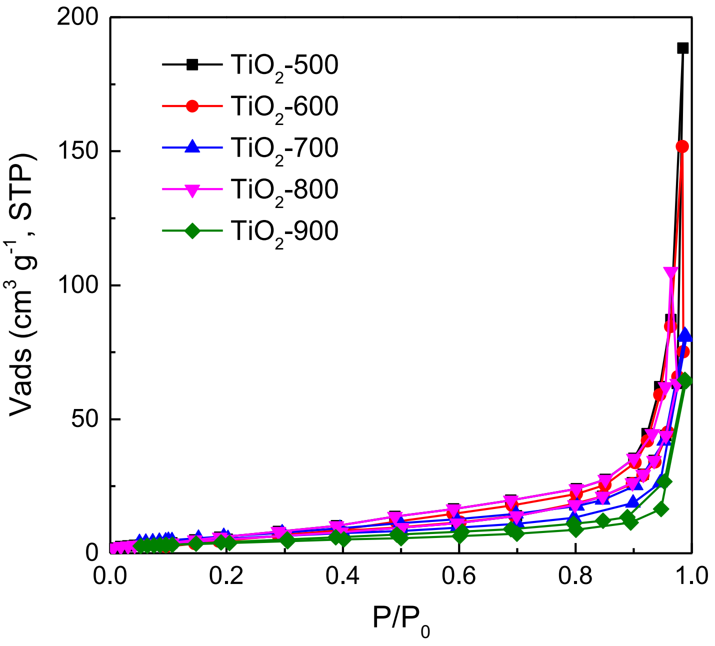

Figure S4. N_2 adsorption-desorption isotherms at −196 °C for TiO_2 materials.

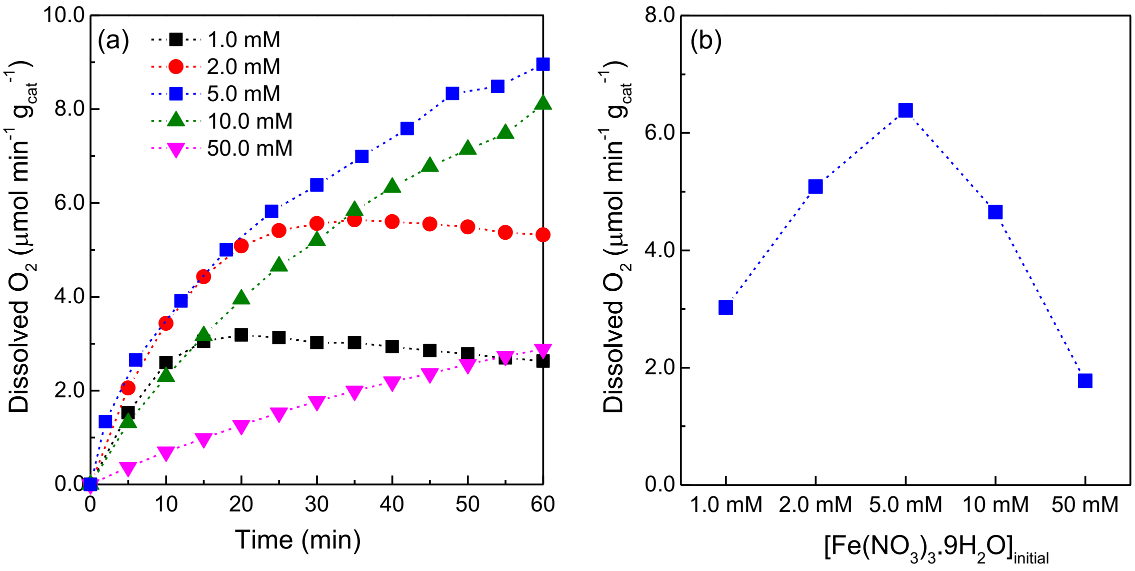

Figure S5. Concentration profiles of sacrificial electron acceptor ($[Fe(NO_3)_3 \cdot 9H_2O]_0$) (a), and concentration of dissolved oxygen after 30 min reaction using TiO_2-700 (b).

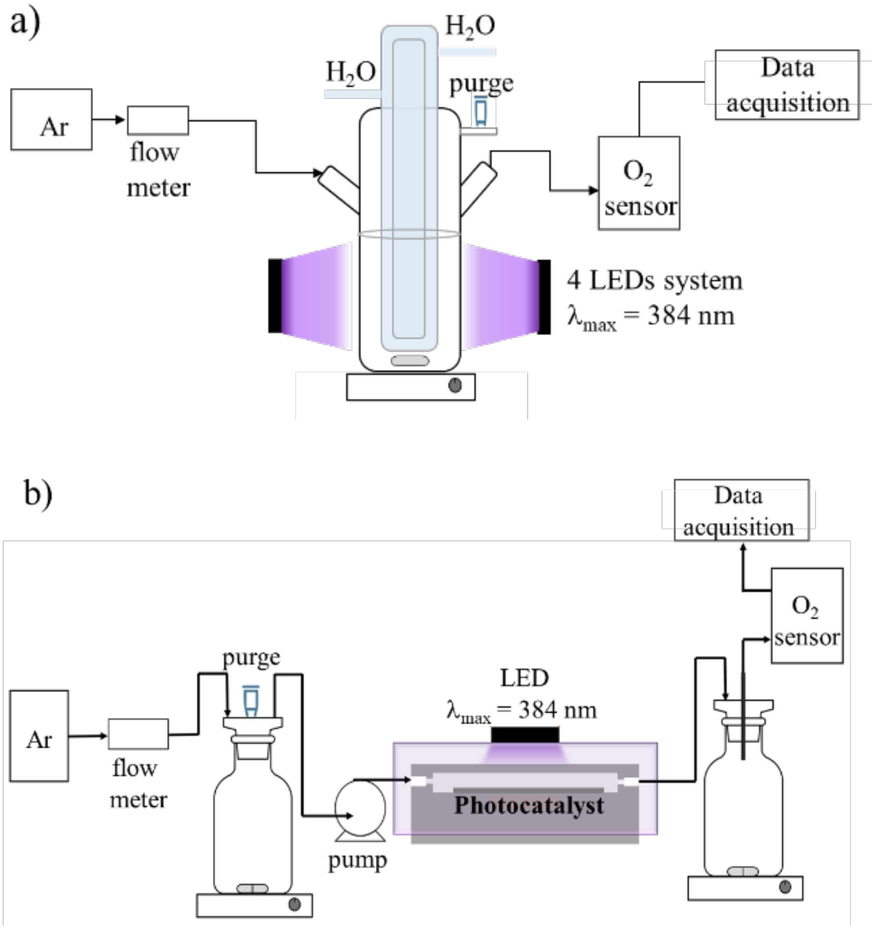

Scheme S1. Photocatalytic experimental set-up under batch reaction mode (a) and a fixed-bed reactor operating under continuous mode (b).
